# Outcome of cerebral embolic protection during transcatheter aortic valve replacement in high-risk patient for stroke

**DOI:** 10.1016/j.xjse.2025.100094

**Published:** 2025-12-17

**Authors:** Chikashi Nakai, Junyi Liu, Nikhil Azhagiri, Andrew Ku, Yuan Haw Wu, Eduardo Danduch, Saeed Tarabichi, Augustin DeLago

**Affiliations:** a Department of Cardiothoracic Surgery, Albany Medical Center, Albany, NY; b Department of Cardiology, Capital Cardiology Associates, Albany, NY

**Keywords:** cerebral embolic protection, stroke, transcatheter aortic valve replacement, transient ischemic attack

## Abstract

**Objectives:**

The sentinel cerebral embolic protection device has been used as prophylaxis of stroke and transient ischemic attack during transcatheter aortic valve replacement. However, there are no absolute criteria guiding cerebral embolic protection device placement. This study aimed to evaluate the impact of cerebral embolic protection on postoperative cerebrovascular events in patients at high risk for stroke.

**Methods:**

Between April 2021 and August 2023, 695 patients underwent transcatheter aortic valve replacement through a transfemoral approach. The cerebral embolic protection device was placed at the beginning of transcatheter aortic valve replacement procedure when 1 of the following criteria was met preoperatively: bicuspid aortic valve, valve-in-valve procedure, or calcium score in computed tomography greater than 1000. Of 695 patients with transcatheter aortic valve replacement, 636 met the criteria. The cerebral embolic protection device was placed in 55% of patients (350/636, cerebral embolic protection group) and not placed in 45% of patients (286/636, non–cerebral embolic protection group) because cerebral embolic protection was not feasible anatomically. A 1:1 propensity score matching was performed using the nearest neighbor method between cerebral embolic protection and non–cerebral embolic protection groups.

**Results:**

A total of 245 pairs were matched in propensity score matching. After matching, postoperative stroke/transient ischemic attack occurred in 0.4% (1/245) in the cerebral embolic protection group and 2.9% (7/245) in the non–cerebral embolic protection group. A significant difference was noted in postoperative stroke/transient ischemic attack between the 2 groups (*P =* .03). Early mortality and other complications did not differ between the 2 groups. In postoperative follow-up, incidence of stroke/transient ischemic attack was similar in 30 days (2.0% vs 1.2%, *P =* .72) and 1 year (0.4% vs 1.2%, *P =* .62). There was no vascular complication at the cerebral embolic protection access site.

**Conclusions:**

Cerebral embolic protection during transcatheter aortic valve replacement might reduce postoperative cerebrovascular events in patients who are high risk for stroke in this patient cohort.

**Figure:**
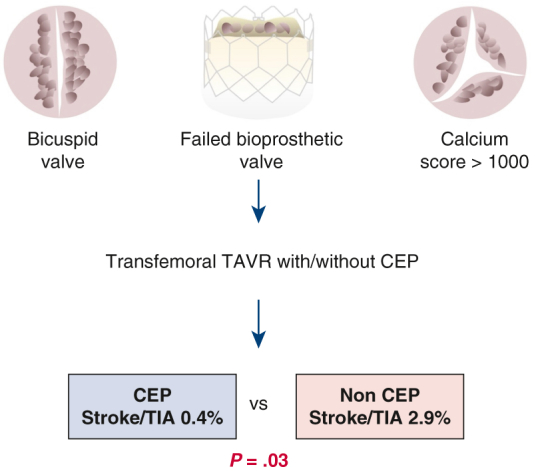
CEP reduced cerebrovascular events in patients high risk for stroke.

**Central Message**

The CEP device is one of the options to reduce postoperative cerebrovascular events in patients undergoing TAVR who are considered high risk for stroke.

**Perspective**

Further study would be required to elucidate more specific patients, such as those with isolated high calcium score, bicuspid valve, and ViV procedure, with the CEP device compared with patients without the protection.

Despite advancements in procedural techniques and device technology in transcatheter aortic valve replacement (TAVR), cerebrovascular events such as stroke and transient ischemic attack (TIA) remain significant complications, with reported periprocedural rates ranging from 2% to 5%.^1^^,^^2^ Cerebral embolic protection (CEP) devices, such as the Sentinel system, have been developed to mitigate this risk by capturing or deflecting embolic material during TAVR.^3^ Although early observational studies and random trials, including the PROTECTED TAVR trial, have demonstrated a trend toward reduced ischemic brain lesions on imaging with CEP use, clinical outcomes such as stroke incidence remain inconsistently reported.^4^^,^^5^ Moreover, current guidelines do not universally endorse routine CEP use due to insufficient evidence supporting its efficacy across diverse patient populations.^6^ Notably, subgroups at heightened embolic risk, such as those with bicuspid aortic valves (BAVs), with extensive aortic calcification (calcium score >1000), or undergoing valve-in-valve (ViV) procedures, may derive greater benefit from CEP; however, data specific to these cohorts are limited.^7^, ^8^, ^9^ This study aimed to evaluate the impact of CEP on postoperative cerebrovascular events in patients undergoing TAVR who exhibited predefined high-risk features for stroke, including BAV anatomy, elevated calcium burden, and ViV procedures.

## Material and Methods

### Patient Selection

**Figure 1:**
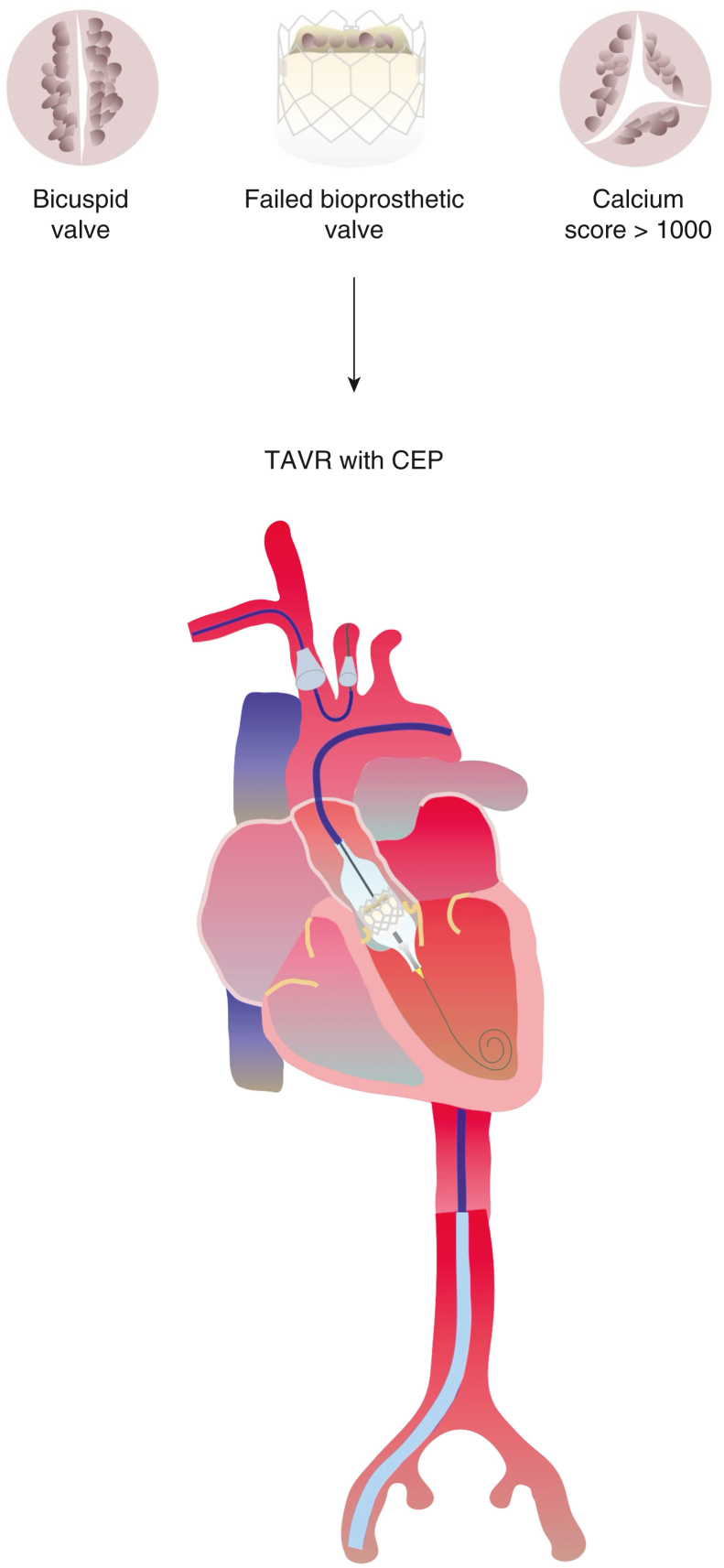
The CEP device was placed through the right radial artery at the beginning of the TAVR procedure when 1 of the following criteria was met preoperatively; patients with BAV, with calcium score in CT more than 1000, or undergoing ViV TAVR procedure. *CEP*, Cerebral embolic protection; *TAVR*, transcatheter aortic valve replacement; *BAV*, bicuspid aortic valve; *ViV*, valve-in-valve.

**Figure 2:**
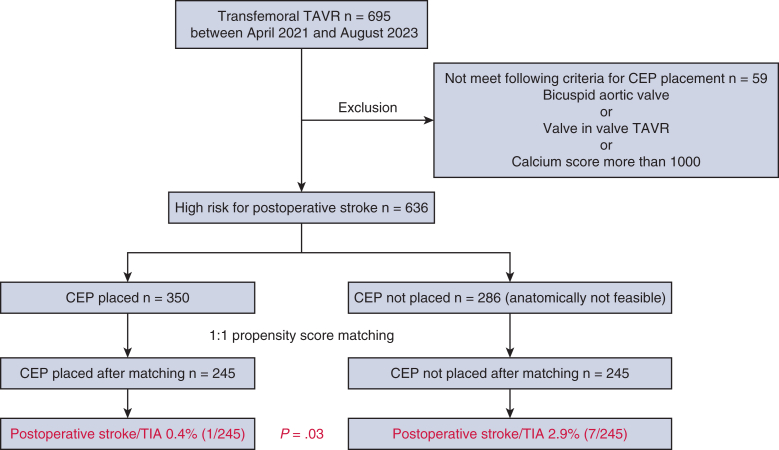
A total of 636 patients undergoing TAVR were considered preoperatively as high risk for stroke. Of 636 patients, the CEP device was placed in 350 and 286 in the non-CEP group. Propensity score matching was performed between the 2 groups (245 pairs matched). Postoperative stroke/TIA was evaluated. *TAVR*, Transcatheter aortic valve replacement; *CEP*, cerebral embolic protection; *TIA*, transient ischemic attack.

This study included consecutive patients who underwent TAVR at Albany Medical Center between April 2021 and August 2023. A total of 695 patients were identified for this study. The CEP device was placed through the right radial artery at the beginning of the TAVR procedure when at least 1 of following criteria was met preoperatively: patients with BAV, calcium score in computed tomography (CT) greater than 1000, or performance of a ViV TAVR procedure (Figure 1). Of the 695 patients undergoing TAVR, 636 met the criteria. The CEP device was placed in 55% of patients (350/636, CEP group) and not placed in 45% of patients (286/636, non-CEP group) due to anatomic infeasibility (Figure 2). Anatomic reasons for CEP nondeployment included severely tortuous vessels between the right radial artery and the innominate artery, bovine aortic arch anatomy, arteriovenous fistula, and hematoma around the right radial artery secondary to recent left heart catheterization. Propensity score matching was performed between the CEP and non-CEP groups, and 245 pairs were matched between the 2 groups. The Institutional Review Board approved the study protocol (No. 6943, March 1, 2024), and the requirement for individual informed consent was waived. A comprehensive chart review was conducted using the electronic health record system at Albany Medical Center. Preoperative, intraprocedural, and postoperative data were collected in a dedicated database prospectively. All patients were followed postoperatively at 30 days and 1 year.

### Study End Points

Early mortality was defined as death occurring within 30 days after TAVR. Postoperative cerebrovascular events were defined as in-hospital postoperative stroke or TIA. Postoperative stroke and TIA were diagnosed by neurologists at Albany Medical Center using CT and magnetic resonance imaging (MRI). Only patients with clinical signs of neurologic events underwent postoperative CT and MRI. The primary end point was the incidence of cerebrovascular event in-hospital, in 30 days, and 1 year. The secondary end point included early mortality and postoperative complications other than cerebrovascular events.

### Statistical Analysis

Categorical variables were described as numbers and percentages, and continuous variables were presented as mean ± SD. Student *t* test was performed for continuous variables that have a normal distribution, and Mann–Whitney *U* test was used for them that do not have a normal distribution. Categorical variables were assessed by chi-square test or Fisher exact test. Propensity score matching was conducted to control for potential confounding variables because of significant differences in preoperative characteristics between the CEP and non-CEP groups. Age, sex, atrial fibrillation, carotid artery stenosis, cerebrovascular disease, cerebrovascular accident, TIA, diabetes mellitus, hypertension, peripheral artery disease, and porcelain aorta were selected as confounding variables based on previous reports. Propensity scores were calculated using a logistic regression model. The model fit was confirmed by the area under the curve (0.67) in the receiver operative characteristic curve. A 1:1 matching was performed using the nearest-neighbor method between the 2 groups. On univariable analyses, the risk factors of in-hospital cerebrovascular event were determined after propensity matching. The predictors of *P* < .15 in univariable analyses were selected for a logistic regression analysis. There were no missing data. Data analyses were performed using SPSS version 29.0.0.0 (IBM).

## Results

Patient characteristics are listed in Table 1. Before propensity matching, the mean age was 76.6 ± 9.1 years in the CEP group and 80.7 ± 8.2 years in the non-CEP group. The non-CEP group was significantly older than the CEP group (*P <* .01). A higher prevalence of male patients was observed in the CEP group (64.0% vs 50.0%, *P <* .01), whereas the number of patients with hypertension was more prevalent in the non-CEP group (64.0% vs 90.2%, *P <* .01). All preoperative characteristics were similar between the 2 groups after propensity matching including age, sex, medical history of hypertension, cerebrovascular disease, carotid artery disease, porcelain aorta, BAV, and the Society of Thoracic Surgeons risk score for isolated aortic valve replacement. Intraoperative and postoperative data are summarized in Table 2. All patients underwent transfemoral TAVR procedure. After propensity matching, 25 patients (10.2%) underwent ViV TAVR in the CEP group and 14 patients (5.7%) underwent ViV TAVR in the non-CEP group (*P =* .07). The type of TAVR valve deployed (balloon expandable vs self-expanding) did not differ between the 2 groups (Table 2).

**Table 1:** Preoperative characteristics

Prematching				Postmatching		
Characteristic	CEP n = 350	Non-CEP n = 286	*P* value	CEP n = 245	Non-CEP n = 245	*P* value
Age, y	76.6 ± 9.1	80.7 ± 8.2	<.01	79.3 ± 8.3	79.6 ± 8.0	.67
Male	224 (64.0)	143 (50.0)	<.01	141 (57.6)	130 (53.1)	.32
Hypertension	224 (64.0)	258 (90.2)	<.01	220 (89.98)	218 (89.0)	.77
Diabetes	114 (32.6)	102 (35.7)	.28	84 (34.3)	83 (33.9)	.92
Atrial fibrillation	112 (32.0)	109 (38.1)	.11	90 (36.7)	91 (37.1)	.93
Cerebrovascular disease	41 (11.7)	32 (11.2)	.84	27 (11.0)	27 (11.0)	1.00
Cerebrovascular accident	32 (9.1)	32 (11.2)	.39	26 (10.6)	28 (11.4)	.77
TIA	21 (6.0)	18 (6.3)	.88	11 (4.5)	17 (6.9)	.24
Carotid artery disease	40 (11.4)	39 (13.6)	.40	29 (11.8)	29 (11.8)	1.00
Peripheral vascular disease	53 (15.1)	57 (19.9)	.11	38 (15.5)	43 (17.6)	.54
Porcelain aorta	27 (7.7)	14 (4.9)	.15	14 (5.7)	14 (5.7)	1.00
BAV	69 (19.7)	41 (14.3)	.07	41 (16.7)	40 (16.3)	.90
Calcium score	2783 ± 1574	2603 ± 1525	.21	2705 ± 1561	2603 ± 1520	.51
Bioprosthetic pure aortic insufficiency	3 (0.9)	1 (0.3)	.63	1 (0.4)	0 (0)	.50
STS risk score, %	3.1 ± 3.2	4.5 ± 4.2	<.01	3.49 ± 3.55	4.1 ± 4.0	.09

Values are mean ± SD or n (%). *CEP*, Cerebral embolic protection; *TIA*, transient ischemic attack; *BAV*, bicuspid aortic valve; *STS*, Society of Thoracic Surgeons.

**Table 2:** Operative procedures and postoperative outcomes

Prematching				Postmatching		
Operative procedures	CEP n = 350	Non-CEP n = 286	*P* value	CEP n = 245	Non-CEP n = 245	*P* value
Transfemoral approach	350 (100)	286 (100)	1.00	245 (100)	245 (100)	1.00
ViV procedure	39 (11.1)	19 (6.6)	.05	25 (10.2)	14 (5.7)	.07
Balloon-expandable valve	350 (100)	283 (99.0)	.09	245 (100)	243 (99.2)	.25
Self-expandable valve	0 (0)	3 (1.0)	.09	0 (0)	2 (0.8)	.25
Postoperative outcomes						
In-hospital cerebrovascular event	4 (1.1)	10 (3.5)	.06	1 (0.4)	7 (2.9)	.03
In-hospital stroke	3 (0.9)	6 (2.1)	.31	1 (0.4)	4 (1.6)	.37
Disabling	1 (0.3)	4 (1.4)	.18	1 (0.4)	2 (0.8)	.50
Nondisabling	2 (0.6)	2 (0.7)	.61	0 (0)	2 (0.8)	.50
Ischemic	3 (0.9)	3 (0.9)	.31	1 (0.4)	4 (1.6)	.37
Conversion to hemorrhagic	0 (0)	0 (0)	N/A	0 (0)	0 (0)	N/A
In-hospital TIA	1 (0.3)	4 (1.4)	.18	0 (0)	3 (1.2)	.25
Permanent pacemaker	34 (9.7)	21 (7.3)	.29	27 (11.0)	17 (6.9)	.11
New dialysis requirement	1 (0.3)	2 (0.7)	.59	0 (0)	1 (0.4)	.50
Vascular surgical intervention for access site	18 (5.1)	21 (7.3)	.25	9 (3.7)	9 (3.7)	1.00
Length of hospital stay, d	2.2 ± 3.8	3.1 ± 4.5	.01	2.3 ± 3.5	2.9 ± 4.6	.12
Early mortality	2 (0.6)	8 (2.8)	.04	2 (0.8)	5 (2.0)	.29
Early mortality + cerebrovascular event	5 (1.3)	15 (5.2)	<.01	2 (0.8)	11 (4.5)	.01
Discharged to home	329 (94.0)	256 (89.5)	.04	227 (92.7)	229 (93.5)	.72
Discharged to rehab/other facility	19 (5.4)	22 (7.7)	.25	16 (6.5)	11 (4.5)	.32
Cerebrovascular event at 30-d follow-up	5 (1.4)	3 (2.4)	.74	5 (2.0)	3 (1.2)	.72
Cerebrovascular event at 1-y follow-up	4 (1.1)	3 (1.0)	.61	1 (0.4)	3 (1.2)	.62

Values are mean ± SD or n (%). *CEP*, Cerebral embolic protection; *ViV*, valve-in-valve; *TIA*, transient ischemic attack.

### Postoperative Cerebrovascular Event

After propensity matching, 1 patient (0.4%) in the CEP group was complicated by an in-hospital cerebrovascular event, and 7 patients (2.9%) were complicated by an in-hospital cerebrovascular event in the non-CEP group (Figure 1). There was a significant difference in the incidence of in-hospital cerebrovascular events after propensity matching between the 2 groups (*P =* .03), whereas that of prematching did not differ (1.1% vs 3.5%, *P =* .06) (Table 2). Each incidence of in-hospital stroke and TIA after matching tended to be lower in the CEP group but did not reach statistical significance (*P =* .37, and *P =* .25) (Table 2). Logistic regression analysis revealed neither age (OR, 1.08, *P =* .15) nor CEP placement (odds ratio, 0.14, *P =* .06) as an independent predictor for in-hospital cerebrovascular event (Table 3). The incidence of new cerebrovascular event at 30-day and 1-year follow-ups after propensity matching was not different between the 2 groups (2.0% vs 1.2%, *P =* .72, 0.4% vs 1.2%, *P =* .62) (Table 2).

**Table 3:** Univariable and logistic regression analysis for in-hospital cerebrovascular event

Risk factors	Adjusted OR	95% CI	*P*
Univariable analysis			
Age	1.07	0.98-1.18	.14
Atrial fibrillation	1.03	0.24-4.34	.97
Carotid artery disease	1.04	0.13-8.64	.97
Peripheral artery disease	1.51	0.30-7.63	.62
CEP placement	0.14	0.02-1.14	.07
Logistic regression analysis			
Age	1.08	0.97-1.20	.15
CEP placement	0.14	0.02-1.13	.06

*OR*, Odds ratio; *CEP*, cerebral embolic protection.

### Patients Complicated by In-hospital Cerebrovascular Event

Preoperative characteristics and postoperative outcomes in patients who were complicated by in-hospital cerebrovascular event are summarized in Table 4. Among 636 patients who underwent TAVR, 9 experienced postoperative stroke and 5 experienced TIA. Eight patients (88.9%) in the stroke group exhibited isolated calcium score greater than 1000, and 5 patients (100%) exhibited isolated calcium score greater than 1000 in the TIA group (Table 4). One patient with both BAV + calcium score greater than 1000 developed postoperative stroke. In the stroke group, 5 patients (55.6%) presented with residual deficits, and 4 died within 30 days of the TAVR procedure. All deaths were neurologic in origin and secondary to postoperative stroke (Table 4). Among the survivors of in-hospital cerebrovascular event, only 1 patient developed a new cerebrovascular event at 30-day follow-up, although no patients experienced new cerebrovascular events at 1-year follow-up (Table 4).

**Table 4:** Outcomes of patients who were complicated by in-hospital cerebrovascular event

Characteristics/events	Stroke n = 9	TIA n = 5
Age, y	81.7 ± 6.1	81.8 ± 7.0
Male	4 (44.4)	2 (40.0)
CEP placement	3 (33.3)	1 (20.0)
Isolated BAV	0 (0)	0 (0)
Isolated calcium score >1000	8 (88.9)	5 (100)
Isolated ViV	0 (0)	0 (0)
BAV + calcium score >1000	1 (11.1)	0 (0)
Length of hospital stay, d	7.4 ± 5.5	2.8 ± 1.8
Early mortality	4 (44.4)	0 (0)
Neurological	4 (100)	0 (0)
Non-neurological	0 (0)	0 (0)
Residual deficit	5 (55.6)	0 (0)
Discharged to home	4 (44.4)	5 (100)
Discharged to rehab/other facility	1 (11.1)	0 (0)
New cerebrovascular event at 30-d follow-up	1 (11.1)	0 (0)
New cerebrovascular event at 1 y follow-up	0 (0)	0 (0)

Values are mean ± SD or n (%). *TIA*, Transient ischemic attack; *CEP*, cerebral embolic protection; *BAV*, bicuspid aortic valve; *ViV*, valve-in-valve.

### Postoperative Outcomes

The early mortality rate after matching was similar between the CEP and non-CEP groups (0.8% vs 2.0%, *P =* .29), although that of prematching was significantly higher in the non-CEP group (0.6% vs 2.8%, *P =* .04) (Table 2). The combined rate of early mortality and cerebrovascular event in the non-CEP group after propensity matching was higher than in the CEP group (0.8% vs 4.5%, *P =* .01) (Table 2). A significant difference was not detected in the incidence of permanent pacemaker placement, new dialysis requirement, vascular surgical intervention for the TAVR sheath access, length of hospital stay, discharged to home, or discharged to rehabilitation or another facility between the 2 groups (Table 2). No vascular complications related to CEP placement were observed.

## Discussion

The findings of this study suggest that the use of CEP during TAVR in high-risk patients was associated with a significant reduction in in-hospital cerebrovascular event compared with unprotected procedures. The observed 0.4% incidence of cerebrovascular events in the CEP group versus 2.9% in the non-CEP group aligned with prior studies demonstrating the potential of CEP to mitigate periprocedural embolic risk.^1^^,^^3^ Notably, this benefit was most pronounced during the immediate postprocedural period, consistent with the hypothesis that cerebral embolization predominantly occurred during valve manipulation and deployment.^4^ The absence of vascular complications related to CEP placement further supported its safety profile in anatomically eligible patients, reinforcing findings from the PROTECTED TAVR trial.^5^ However, the lack of significant differences in rates of cerebrovascular event at 30 days and 1 year underscored the transient nature of CEP's protective effect, which likely targeted acute embolic events rather than long-term cerebrovascular risk. The results were consistent with prior evidence suggesting that most TAVR-related strokes occurred within 48 hours of the procedure.^2^

This study's focused on high-risk subgroups—patients with BAV or elevated calcium scores (>1000) or those undergoing ViV procedures—addressed a critical gap in existing data. These cohorts were inherently predisposed to embolic complications due to complex anatomy and heavy calcification.^7^, ^8^, ^9^ Our findings suggested that CEP might offer a targeted benefit in these populations, potentially refining patient selection beyond current guideline recommendations, which remained equivocal on routine CEP use.^6^

Notably, the combined end point of early mortality and cerebrovascular events was significantly higher in the non-CEP group compared with the CEP group (4.5% vs 0.8%, *P =* .01), emphasizing the additive burden of periprocedural embolic complications. Prior studies have demonstrated that stroke after TAVR not only increases short-term mortality but also contributes to prolonged hospitalization and adverse recovery trajectories.^10^ In our cohort, 4 of the 9 patients (44.4%) who developed perioperative stroke died within 30 days, all due to neurologic causes, highlighting the severe and often fatal nature of these complications. No deaths were due to non-neurological causes in the stroke group.

Beyond mortality, stroke was frequently associated with lasting morbidity: Five of the 9 patients (55.6%) who experienced stroke were discharged with residual neurologic deficits. This finding aligned with prior studies linking post-TAVR stroke to prolonged functional impairment and reduced independence.^11^ Accordingly, even a small number of cerebrovascular events may yield a disproportionately large burden, both for patients and healthcare systems.

A key anatomic risk factor identified in this study was an isolated aortic valve calcium score greater than 1000, which was present in 88.9% of stroke cases and in all TIA cases. These findings suggest that severe valve calcification may independently predispose patients to cerebral embolization during TAVR. Prior imaging studies have demonstrated a direct relationship between high calcific burden and both the quantity of embolic debris captured during valve deployment and the occurrence of new ischemic brain lesions on postprocedural MRI.^12^ This highlights the importance of considering CEP in patients with heavy calcification, even in the absence of other procedural or anatomic risk features. These findings advocate for integrating calcium scoring into procedural planning. Patients with isolated calcium scores greater than 1000 may represent an underrecognized subgroup at heightened risk for embolic complications and could be considered prime candidates for routine CEP use. Emerging evidence suggests that the calcium-driven risk model may outperform conventional surgical or clinical scores in predicting periprocedural embolization during TAVR.^13^ Collectively, these findings highlight CEP as a critical tool for mitigating early cerebrovascular events and mortality in high-risk patients undergoing TAVR.

### Study Limitations

First, the retrospective, single-center design introduced risks of selection bias, particularly because CEP placement was contingent on anatomic feasibility. Second, the number of patients who were complicated by postoperative cerebrovascular events was relatively small. As a result, the multivariable analysis evaluating predictors of postoperative cerebrovascular event was underpowered. Third, the population of patients who did not receive CEP was high because of anatomic constraints. Most cases were due to severely tortured vessels, and the decision to abort the CEP placement was made intraoperatively at the operator's discretion. Fourth, there might be an additional risk factor for postoperative stroke that was not evaluated preoperatively in this study, such as aortic wall thrombus.^14^ Fifth, although peripheral artery disease was included as a variable, the severity of peripheral artery disease was not recorded in the registry. However, peripheral artery disease was not identified as a risk factor for postoperative stroke or TIA in this patient cohort.

## Conclusions

The CEP device during TAVR might reduce postoperative cerebrovascular events in patients with high risk for stroke including those with BAV or high calcium score, and those undergoing ViV procedure. An early mortality rate was high among patients who developed in-hospital stroke. The placement of cerebral protection device was performed safely without complications in this patient cohort.

## Conflict of Interest Statement

The authors reported no conflicts of interest.

The *Journal* policy requires editors and reviewers to disclose conflicts of interest and to decline handling or reviewing manuscripts for which they may have a conflict of interest. The editors and reviewers of this article have no conflicts of interest.
